# Rapid and Scalable Synthesis, Structure, and Physical Properties of Solid-Solution Layered Titanium Niobium Carbosulfide “Ti_*x*_Nb_2–*x*_SC”

**DOI:** 10.1021/acsomega.6c08457

**Published:** 2026-09-23

**Authors:** Ahmad Majed, Asaf Pesach, Anna Eyal, Amit Keren, Mina Saleh, Kaitlyn Prenger, El’ad N. Caspi, Maxim Avdeev, Michael Naguib

**Affiliations:** † Department of Physics and Engineering Physics, 5783 Tulane University, New Orleans, Louisiana 70118, United States; ‡ Physics Department, Nuclear Research Centre − Negev, P.O. Box 9001, Beer-Sheva 84190, Israel; § Physics Department, Technion, Haifa 32000, Israel; ∥ Australian Centre for Neutron Scattering, Australian Nuclear Science and Technology Organisation, Locked Bag 2001, Kirrawee DC, NSW 2232, Australia; ⊥ School of Chemistry, University of Sydney, Sydney, NSW 2006, Australia

## Abstract

We demonstrate rapid, scalable synthesis of solid-solution layered transition metal carbosulfides, Ti_*x*_Nb_2–*x*_SC (0 ≤ *x* ≤ 2). Thermogravimetric analysis confirmed superior thermal stability of Ti_2_SC vs Nb_2_SC; oxidation of Nb_2_SC in air (750 °C) produced an accordion-like sulfur-bearing niobium oxide. Room-temperature X-ray diffraction and temperature-dependent neutron diffraction revealed linear contraction of lattice parameters with increasing Ti content. Strong anisotropic thermal expansion behavior arises from metallic basal planes vs nonmetallic M–S bonds, with Nb_2_SC showing larger volumetric expansion than Ti_2_SC. Specific heat measurments confirm that Ti_2_SC is stiffer than Nb_2_SC. In both thermal expansion and specific heat, nonmonotonic behaviors are linked to the critical role of Ti/Nb mixing. SQUID measurements show diamagnetic shielding in high-Nb samples, which, lacking an anomaly in specific heat, is ascribed to trace, percolating superconducting impurities rather than bulk superconductivity.

## Graphical abstract

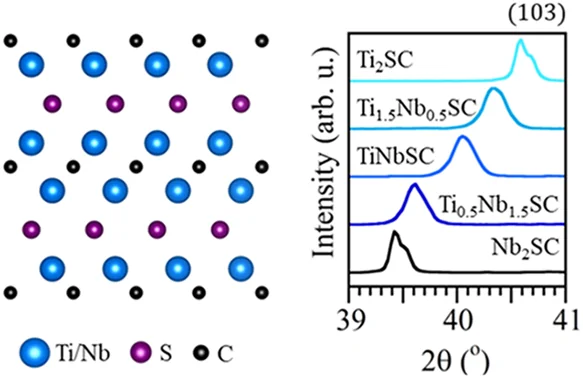

## 1 Introduction

Layered ternary transition metal carbides, initially known as Hagg phases, were first reported in 1960 by Rohde and Kudielka,^1^ followed by the discovery of a substantial number of new materials belonging to this phase by Hans Nowotny and co-workers.^2–4^ In 1998, Barsoum and El-Raghy presented their research on Ti_3_SiC_2_.^5^ Barsoum later introduced the MAX phases in 2000, describing them as hexagonal layered ternary transition metal carbides and nitrides with space group *D*_6*h*_^4^–*P*6_3_ */mmc* (Figure 1a). These compounds have the chemical formula M_*n*+1_AX*_n_*, where M represents an early transition metal, A represents elements from group IIIA (Al, Ga, In), group IVA (Si, Ge, Sn), group VA (P, As), and group VIA (S), and X denotes C and/or N.^6^ Through selective etching of the A layer from MAX phases, M_*n*+1_X*_n_* T_*x*_ compounds are formed, belonging to the 2D MXene family, with T_*x*_ representing surface terminations such as H, OH, and F.^7^

**Figure 1:**
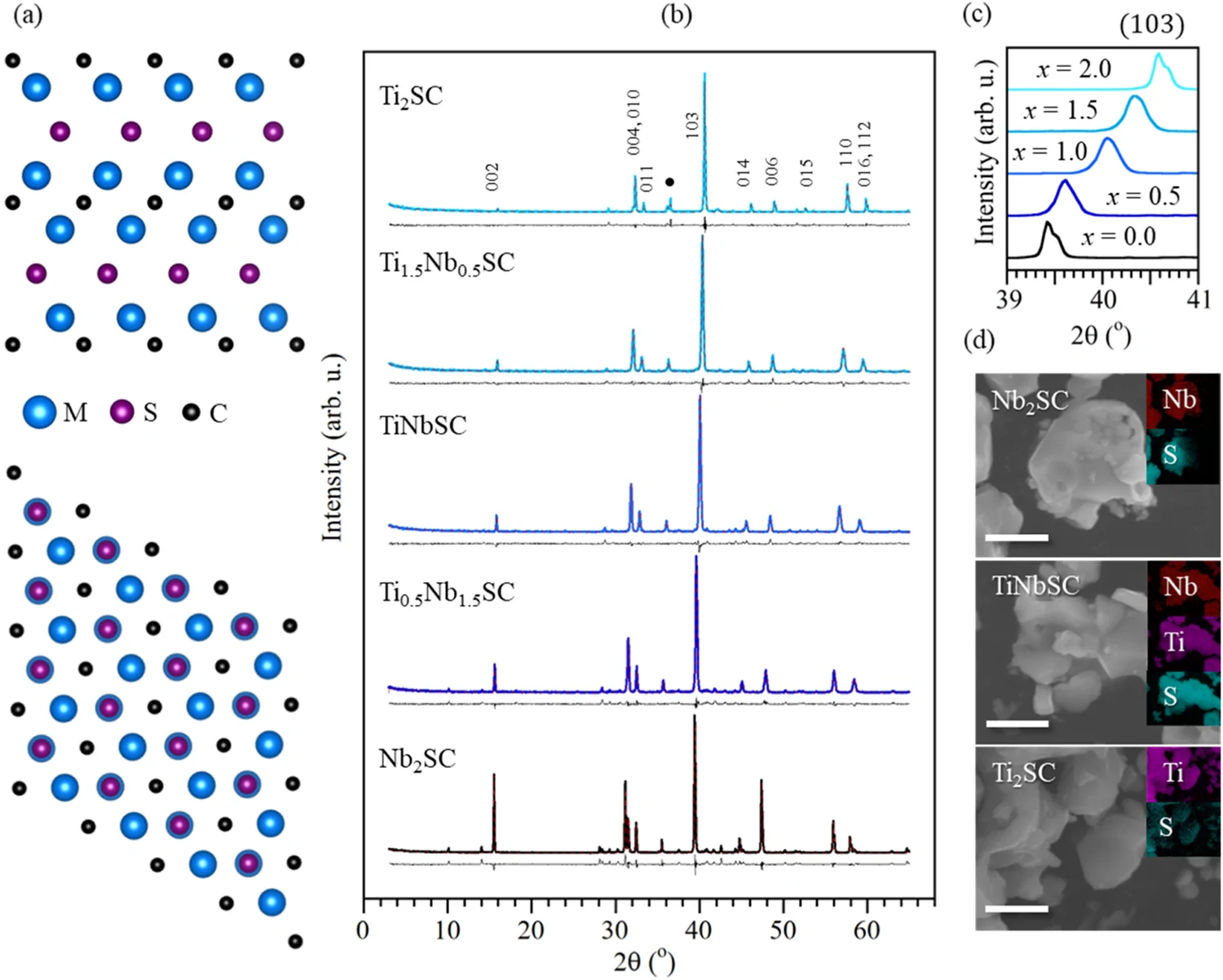
(a) Schematic for the atomic arrangement of MAX phases studied here. (b) XRD data of Ti_*x*_Nb_2–*x*_SC (for *x* = 0, 0.5, 1, 1.5, 2) and Rietveld refinement plot showing calculated profiles in colored solid lines. Below each plot, the difference between the observed and calculated profiles can be found. (c) XRD plot of (103) peak shift dependency on *x*. (d) SEM images of Nb_2_SC, TiNbSC, and Ti_2_SC along with EDS mapping showing Nb, Ti, and S elements. The scale bar represents 5.0 μm. The closed black circle in (b) represents the main diffraction peak of the secondary phase titanium carbide.

Layered carbosulfide MAX phases have sulfur as the A element (group VIA), which has a higher electronegativity compared to elements from group IIIA (Al, Ga, In) commonly found in other MAX phases. The *c*-lattice constant of Nb_2_SC is significantly lower than that of Nb_2_AlC, Nb_2_GaC, and Nb_2_InC, indicating a stronger bond between Nb and S.^8^ This stronger M–A bond explains why MXenes have not been synthesized from carbosulfide MAX phases thus far. However, metastable Nb_2_S_2_C has been reported, with weak out-of-plane S–S bonds in its atomic stacking of S–Nb–C–Nb–S.^9–12^ Nb_2_S_2_C demonstrates potential exfoliability with a predicted cleavage energy of 0.38 J·m^–2^.^12^ Its synthesis involved selective etching of Cu from Cu_0.67_Nb_2_S_2_C, which was obtained by heating Nb_2_SC+S+Cu.^9,10^

Layered transition metal carbosulfides offer intriguing applications. Notably, superconducting behavior was reported for Nb_2_SC_0.9_ at *T*_c_ = 5 K,^13^ Cu_0.67_Nb_2_S_2_C at *T*_c_ = 7.2 K,^14^ and Nb_2_S_2_C at *T*_c_ = 7.6 K.^14^ Ti_2_SC displays exceptional stability under compression up to 47 GPa without phase transformation.^15^ Its room-temperature thermal conductivity is ≈60 W/m K, surpassing most of the reported MAX phases, while its Debye temperature, previously reported in the range ∼745–765 K, is among the highest in those phases as well.^16^ First-principles density functional theory (DFT) studies revealed that increasing the Nb content in Ti_*x*_Nb_2–*x*_SC solid solution increases bulk modulus and Poisson’s ratio, but detrimentally affects Young’s modulus and Debye temperature.^17^

Historically, the low boiling point of chalcogens relative to the formation temperatures of transition metal carbo-chalcogenides has necessitated a closed heating environment, like vacuum-sealed quartz tubes. This has limited the scalability (<1 g), heating temperature (<1200 °C), and required extended heating durations (>6 days).^10^ However, in 2015, Ti_2_SC was successfully synthesized using iron sulfide instead of elemental sulfur, followed by acid treatment to remove the iron byproduct.^18^ The higher melting point of transition metal sulfides allowed for (1) heating in an ambient-pressure inert environment, (2) higher-temperature heating (up to 1700 °C), (3) significantly shorter required heating durations, and (4) substantial scaling of the reaction. Previous reports on carbo-chalcogenides include Nb_2_SC,^13,19^ Ti_2_SC,^15,16,18,20^ (Ti,Zr,Hf)_2_SC,^21^ and Zr_2_SeC,^22^ as M_2_AX transition metal carbo-chalcogenides, but iron sulfide was specifically employed only for Ti_2_SC synthesis. Furthermore, Nb_2_S_2_C has been synthesized using Nb_2_SC, suggesting the possibility of other M_2_AX phases producing M_2_A_2_X. To the best of our knowledge, scalable synthesis of Ti_*x*_Nb_2–*x*_SC solid solutions with 0 ≤ *x* ≤ 2 has not been previously reported. In this work, we report a comprehensive study of the temperature-dependent structural and physical properties, as well as the stability of the Ti_*x*_Nb_2–*x*_SC solid solutions (*x* = 0, 0.5, 1, 1.5, 2) synthesized using the rapid and scalable route described above.

## 2 Synthesis and Characterization

### 2.1 Solid-State Synthesis

The precursors for this study were mixed in various molar ratios listed in Table S1 to generate different stoichiometries of Ti_*x*_Nb_2–*x*_SC. Titanium (Ti, 99.5%, <45 μm), titanium carbide (TiC, 99.5%, ∼2 μm), niobium (Nb, 99.8%, <45 μm), and niobium carbide (NbC, 99%, <10 μm) were purchased from Alfa Aesar and used as received. Iron­(II) sulfide (FeS, 99%, 60–200 μm) was purchased from Strem. The chemicals were combined in a high-density polyethylene jar inside an argon-filled glovebox, sealed with parafilm, and then mixed in a Turbula T2F mixer at 56 rpm for 3 h. The resulting powders were transferred in the glovebox and pressed into 1-in. diameter pellets (up to 50 g each), placed in an alumina crucible, and heated from room temperature to 1550 °C at a ramp of 10 °C/min in an alumina tube furnace operated within a chemical fume hood for safety. The temperature was held for 2 h before allowing the samples to cool to room temperature. The heating, holding, and cooling steps were carried out under a continuous flow of argon gas.

### 2.2 Iron Removal

To eliminate iron byproduct after synthesis from the samples, the sintered pellets were ground to particles smaller than 44 μm and added to aqueous hydrochloric acid (12 M HCl) in a ratio of 1 g of powder per 20 mL of HCl solution. The solution was stirred using a Teflon-coated magnetic stir bar at 50 °C for 24 h. The sample was subsequently washed with deionized (DI) water to remove impurities and neutralize the solution to pH ∼ 7. The solution was vacuum-filtered overnight using a Whatman filter paper and a vacuum pump. Residual moisture was removed by heating it at 110 °C under vacuum for 4 h.

### 2.3 Measurements

The crystal structures of the investigated samples were analyzed using a Rigaku D/Max-2200 X-ray diffractometer with a Cu Kα source. Scans were performed with a 2θ step size of 0.02° and a sweep rate of 1°/min. Scanning electron microscope (SEM) images were obtained using a Hitachi S-4800 microscope at an accelerating voltage of 3 kV. Chemical compositions were confirmed through energy-dispersive X-ray spectroscopy (EDS) analysis with an INCA-Hitachi S-3400 microscope at an accelerating voltage of 30 kV. These measurements were done at Tulane University, USA.

Low intensity neutron powder diffraction (NPD) measurements on Ti_*x*_Nb_2–*x*_SC (for *x* = 0, 0.5) were conducted on the KANDI-II powder diffractometer^23^ at the Israeli Research Reactor II (IRR-II) at the Nuclear Research Centre–Negev (NRCN), Israel, at 10 K, and 300 K by using a wavelength of λ = 2.47(1) Å.^24^ Temperature-dependent NPD on Ti_*x*_Nb_2–*x*_SC (*x* = 0, 1, 2) were conducted at the OPAL reactor (ANSTO, Lucas Heights, Australia) from 4 to 320 K, 360 and 370 K, respectively, with 1 K step, on the Wombat diffractometer^25^ by using neutron wavelength of 1.5411 Å. High-resolution NPD measurements on Nb_2_SC were conducted at 3, 6, 11, 50, and 100 K on the Echidna diffractometer^26^ using a neutron wavelength of 2.4395 Å. High-intensity NPD measurements on Nb_2_SC were conducted at 4, 6, 9, and 12 K on the Wombat diffractometer (ANSTO, Australia) using a neutron wavelength of 2.4147 Å.^25^ Different diffractometers were used for the search for spontaneous magnetic order, magnetic transition, and crystallographic characterization as a function of temperature. NPD patterns were analyzed using the Rietveld refinement method^27^ with FullProf software,^28^ while the X-ray diffraction (XRD) patterns were analyzed with Topas software.^29^ Low-temperature measurements were carried out using a closed-cycle helium refrigerator.

Magnetization measurements on Ti_*x*_Nb_2–*x*_SC (*x* = 0, 0.5, 1, 2) samples were obtained at zero-field-cooled (ZFC)–field-cooled (FC) temperature scans under an applied magnetic field of H = 40–50 Oe. Measurements were conducted on the Quantum Design MPMS3 SQUID magnetometer at the Quantum Material Research Center at the Institute of Technology (Technion), Israel. Field-scan magnetization measurements at constant temperatures were also carried out using H between 0 and 70 kOe at selected temperatures between 2 and 400 K. Magnetization measurements were made in search for spontaneous magnetism and superconductivity. These measurements served as a sensitive probe for the detection of iron and iron-based impurities due to this element’s strong magnetic interaction.

Heat capacity measurements on Ti_*x*_Nb_2–*x*_SC (*x* = 0, 0.5, 1, 1.5, 2) samples were conducted as a function of temperature in the range 2–300 K on the Quantum Design PPMS Dyna-Cool instrument at the Quantum Material Research Center at the Technion, Israel.

## 3 Results and Discussion

### 3.1 Ambient Structure Properties

XRD patterns of Nb_2_SC, Ti_0.5_Nb_1.5_SC, TiNbSC, Ti_1.5_Nb_0.5_SC, and Ti_2_SC after iron removal are readily described by calculated crystal structure profiles based on the *P*6_3_/*mmc* [194] hexagonal space group (Figure 1b,c). Increasing *x* from 0.0 to 2.0 resulted in a noticeable shift of the (103) peak from 39.4° to 40.6°, respectively (Figure 1c), indicating a monotonic decrease in lattice parameters of a single phase when Nb is gradually substituted by Ti. This supports the successful formation of Ti_*x*_Nb_2–*x*_SC solid solutions, as no separate peaks for Nb_2_SC and Ti_2_SC are observed. Furthermore, SEM images of Nb_2_SC, TiNbSC, and Ti_2_SC, along with an EDS map illustrating the distribution of Nb, Ti, and S elements, show a uniform distribution of Nb and Ti in TiNbSC on the micro level (Figure 1d). Finally, an additional EDS plot shows no sign of Fe in the sample after scaling up the iron removal step from 1 to 15 g per batch (Figure S1). Rietveld analysis of the XRD data shows a monotonic decrease in the lattice parameters as the Ti content, *x*, is increased (Table 1 and Figure S2). When relevant, the Ti/Nb occupancy ratio is determined and found to agree with the nominal ratio used in the synthesis of the samples.

**Table 1:** Lattice Parameters of Ti_*x*_Nb_2–*x*_SC Determined by Rietveld Analysis of XRD and NPD Data^a^

phase	source	temperature (K)	*a* (Å)	*c* (Å)	*c*/*a*	Nb/Ti occ.	*R*_B_ (%)
Nb_2_SC	XRD	300	3.29720(7)	11.5471(3)	3.5021(1)	1.0/0.0	9.451
	NPD (KANDI-II)	300	3.2928(2)	11.541(2)	3.5049(6)	1.0/0.0	11.470
	NPD (Echidna)	3	3.29062(2)	11.5284(1)	3.50341(5)	1.0/0.0	5.190
	NPD (Wombat)	4	3.28461(9)	11.5076(4)	3.5035(2)	1.0/0.0	10.580
	NPD (KANDI-II)	10	3.2926(3)	11.539(2)	3.5045(7)	1.0/0.0	12.350
Ti_0.5_Nb_1.5_SC	XRD	300	3.2875(2)	11.4184(9)	3.4733(3)	1.8(1)/0.2(1)	2.623
	NPD (KANDI-II)	300	3.2854(2)	11.427(1)	3.4781(4)	1.8(1)/0.2(1)	6.874
	NPD (KANDI-II)	10	3.2831(2)	11.410(2)	3.4754(6)	1.8(1)/0.2(1)	7.096
TiNbSC	XRD	300	3.2567(2)	11.3087(8)	3.4724(3)	1.04(6)/0.96(6)	4.478
Ti_1.5_Nb_0.5_SC	XRD	300	3.2336(2)	11.2493(8)	3.4789(3)	0.51(4)/1.49(4)	3.201
Ti_2_SC	XRD	300	3.2055(2)	11.1898(6)	3.4908(3)	0.0/1.0	1.203

a *R*_B_ is the Bragg R factor of the main phase as provided by the TOPAS code^29^ for XRD and the FULLPROF code^28^ for NPD.

According to the XRD data, the main MAX phase’s content in the Nb_2_SC sample is ∼90 wt %. The main MAX phase’s content in the other Ti_*x*_Nb_2–*x*_SC samples (*x* = 0.5, 1, 1.5, 2) increases to ∼96–98 wt %. In all of the Nb-containing samples, the existence of the antiferromagnetic and possibly superconducting FeS,^30–32^ and paramagnetic/diamagnetic FeS_2_ ^33,34^ (∼0.5–2.5 wt %) could not be ruled out, while an indication of FeS existence (<0.5 wt %) is found in Ti_2_SC as well. In Nb_2_SC, the complex magnet Fe_2_Nb^35^ is found in relatively large amounts (∼5 wt %) together with traces of NbC.^36^ Solid solutions of (Ti_1–*y*_Nb_*y*_)C are found in small amounts (∼0.5–1 wt %) for Ti_*x*_Nb_2–*x*_SC samples (*x* = 0.5, 1, 1.5), and TiC^36^ (∼1.5 wt %) is found in Ti_2_SC. These results do not agree with our EDS findings, possibly due to the limitation of EDS analysis to quantitative chemical analysis of the surface, while XRD provides information about the bulk material.

NPD measurements of Nb_2_SC and Ti_0.5_Nb_1.5_SC at 300 K on KANDI-II are well described by the calculated hexagonal crystal structure, based on the *P*6_3_/*mmc* space group (Figure 2). This is in agreement with the XRD data described above. Refined lattice parameters from Rietveld analysis of NPD data (Table 1 and Figure S2) are consistent with the refined values obtained from XRD data.

**Figure 2:**
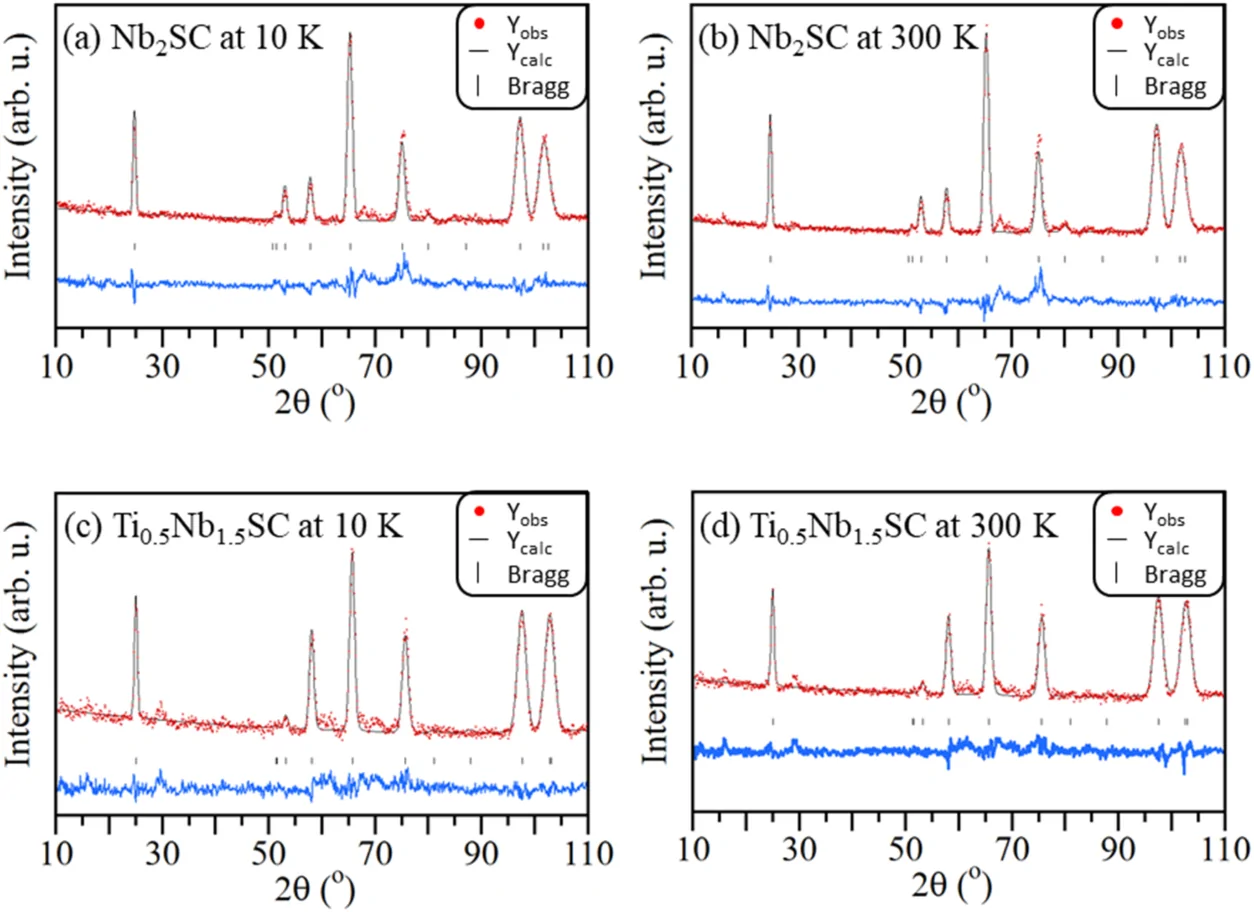
NPD profile of Ti_*x*_Nb_2‑x_SC (a, b) Nb_2_SC and (c, d) Ti_0.5_Nb_1.5_SC. The measurements were recorded at two different temperatures: (a, c) 10 K and (b, d) 300 K. The observed NPD profiles are plotted (red symbols) as a function of scattering angle, 2θ. The Rietveld refined profiles (black lines) fit the observed results. The differences between the observed and refined patterns are shown in the lower part of each figure (blue lines). The peak positions of the main phase and impurities are shown as tick marks.

Not unlike what is found in XRD, the main MAX phase fraction in both Nb_2_SC and Ti_0.5_Nb_1.5_SC, determined by NPD, is ∼94 wt %. However, in the NPD results, additional impurities are found: the paramagnetic NbS,^37^ and superconducting Nb_2_S_2_C.^9^ The much larger powder content, measured in the NPD measurements, compared to XRD measurements, as well as the different sensitivities between neutron and X-ray scattering from matter, may explain these differences in impurities detection. It is important to note that both the main phase and the impurities were consistent across samples from two different batches, as shown in the low-temperature NPD measurements described below, which were performed on different diffractometers. For Nb_2_SC, the refined lattice parameters determined by XRD were *a* = 3.29720(7) Å and *c* = 11.5471(3) Å, consistent with previous reports.^19^ In the case of Ti_2_SC, the refined lattice parameters were *a* = 3.2055(2) Å and *c* = 11.1898(6) Å, similar to literature values of *a* = 3.2051 Å and slightly different from *c* = 11.2636 Å,^16^ but still within a reasonable margin of ∼0.6%. Both the *a* and *c* parameters showed a monotonic decrease with increasing the Ti content (Figure S2), likely due to the smaller atomic size of Ti vs Nb.

It is worth noting that 50 g batches were synthesized for each compound, and several specimens of various sizes were collected from each batch after the powder was thoroughly mixed. Therefore, each specimen is representative of the entire 50 g batch, as demonstrated by the consistent XRD and NPD results obtained from multiple specimens from the same batch.

### 3.2 Structural Properties at Elevated Temperatures

The thermal stability of Ti_*x*_Nb_2–*x*_SC in air was investigated using TGA, as shown in Figure 3a. Since the reaction path occurred over a range of temperatures, the temperature values in this study were recorded in reference to the midpoints of their reactions. Nb_2_SC and Ti_0.5_Nb_1.5_SC witnessed mass gains of 15.2% and 14.6% at ∼475 °C and ∼480 °C, respectively. On the other hand, Ti_1.5_Nb_0.5_SC and Ti_2_SC exhibited higher oxidation temperatures of ∼657 °C and ∼650 °C with mass gains of 10.9% and 10.6%, respectively. TiNbSC showed an intermediate reaction temperature at ∼570 °C with a mass gain of 14.3%. To understand the resulting structure after heating in air, Ti_*x*_Nb_2–*x*_SC samples were heated in air to 750 °C for 30 min. XRD patterns were collected and plotted in Figure 3b. The crystalline oxidation products are transition metal oxides: anatase TiO_2_ for Ti_2_SC, Nb_2_O_5_ for Nb_2_SC, and a mixture of TiNb_2_O_7_, anatase TiO_2_, and Nb_2_O_5_ for the solid solutions Ti_*x*_Nb_2–*x*_SC (*x* = 0.5–1.5), with no sulfur-containing crystalline phases detected by XRD. It is therefore reasonable to assume that most of the S and C are released as SO_2_ and CO_2_, respectively. However, as evidenced by the localized EDS mapping (see below), the rapid oxidation kinetics of the physical system permit the partial retention of sulfur within the resultant oxide matrices. Interestingly, the color of Nb_2_SC powder after oxidation was orange, as shown in the inset of Figure 3a. Given that Nb_2_O_5_ is white in color, this suggests sulfur might be entrapped in the oxidized material. Sulfur-doped transition metal oxides can potentially be used in interesting applications, such as enhanced electrocatalytic activity.^38^ EDS analysis revealed no sulfur for all samples at low magnification. However, at high magnification, sulfur was detected in the Nb_2_SC sample, with a Nb:S atomic ratio of 2.00:0.16. While oxygen vacancies in pure niobium oxides typically induce gray, blue, or brown color centers,^39,40^ the oxidized Nb_2_SC powder exhibited a distinct orange hue. This macroscopic optical shift, coupled with the localized EDS signal, supports the retention of sulfur within the oxidized matrix. Because EDS cannot differentiate between atomic sulfur doping within the Nb_2_O_5_ lattice and the entrapment of unreacted nanoscale sulfide/sulfate phases, further high-resolution chemical analysis (e.g., X-ray photoelectron spectroscopy) is required to determine the precise chemical coordination of the retained sulfur. The SEM images of the oxidized samples in Figure 3c depict an interesting accordion-like layered morphology for Nb_2_SC and TiNbSC samples. Ti/Nb oxides with layered morphology were previously reported for energy storage applications with a relatively longer procedure beginning with the synthesis of the TiNbAlC MAX phase, followed by HF-etching into MXene, and finally controlled oxidation.^41^ This oxidation pathway may provide a simple route to sulfur-modified layered mixed oxides directly from carbosulfide MAX precursors.

**Figure 3:**
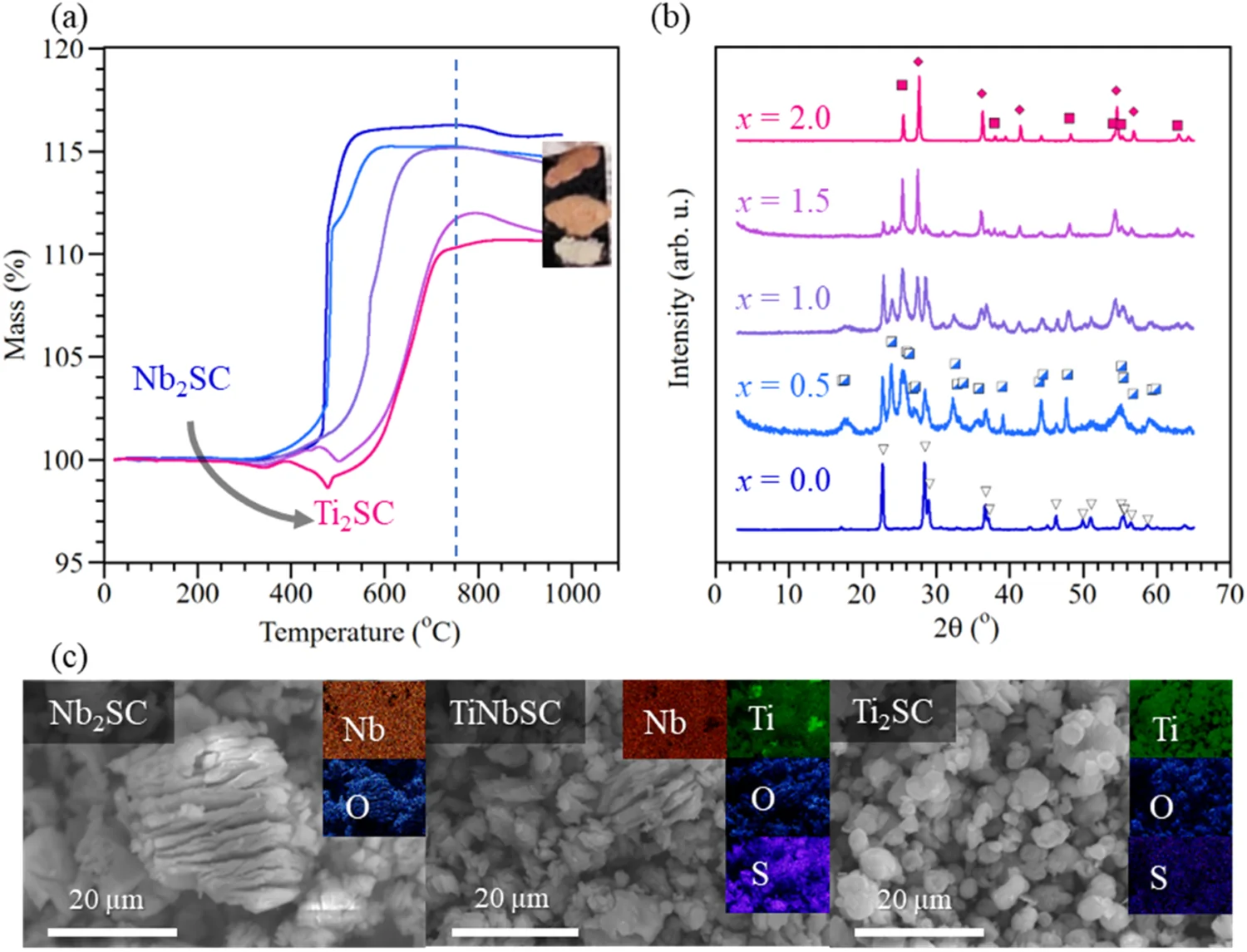
Oxidation of Ti_*x*_Nb_2–*x*_SC. (a) Thermogravimetric analysis (TGA) of Nb_2_SC, Ti_2_SC, and their solid solutions. The inset shows the resultant powders after heating. (b) XRD and (c) SEM with EDS mapping of Ti_*x*_Nb_2–*x*_SC powders in air at 750 °C for 30 min. The dashed line in (a) marks the 750 °C temperature on the TGA graph. The XRD peaks were assigned to Nb_2_O_5_ (PDF#00-027-1003) for [▽], TiO_2_ anatase (PDF#00-064-0863) for [pink diamond solid], TiO_2_ rutile (PDF#01-087-0710) for [pink box solid], and TiNb_2_O_7_ (PDF#00-039-1407) for [blue square lower right solid].

### 3.3 Physical and Structural Properties at Low Temperatures

The temperature-dependent ZFC-FC magnetic susceptibility of Ti_*x*_Nb_2–*x*_SC (*x* = 0, 0.5, 1, 2) shows a general increase upon cooling, with various anomalies indicative of magnetic or electronic transitions (Figure 4). Coalescence of the ZFC and FC curves for *x* = 0, 0.5, and 1 in the temperature range of 180–200 K suggests the existence of ferromagnetic interactions in these samples below room temperature. For *x* = 2, this probably happens only above the highest measurement point of ∼400 K. The ZFC curves of *x* = 0 and 0.5 show a sharp decrease at temperatures of 3.6 and 2.9 K, respectively, reminiscent of superconductivity (Figure 4, inset). However, no absolute negative susceptibility is observed. Crucially, our low-temperature specific heat measurements (see below) reveal no corresponding lambda anomaly at these temperatures. The absence of a bulk thermodynamic phase transition confirms that the main MAX phase is not a bulk superconductor. Instead, this partial diamagnetic shielding likely arises from trace, interconnected superconducting impurities (such as the Nb_2_S_2_C phase detected in our NPD analysis) whose actual volume fraction is too small to be detected in specific heat.

**Figure 4:**
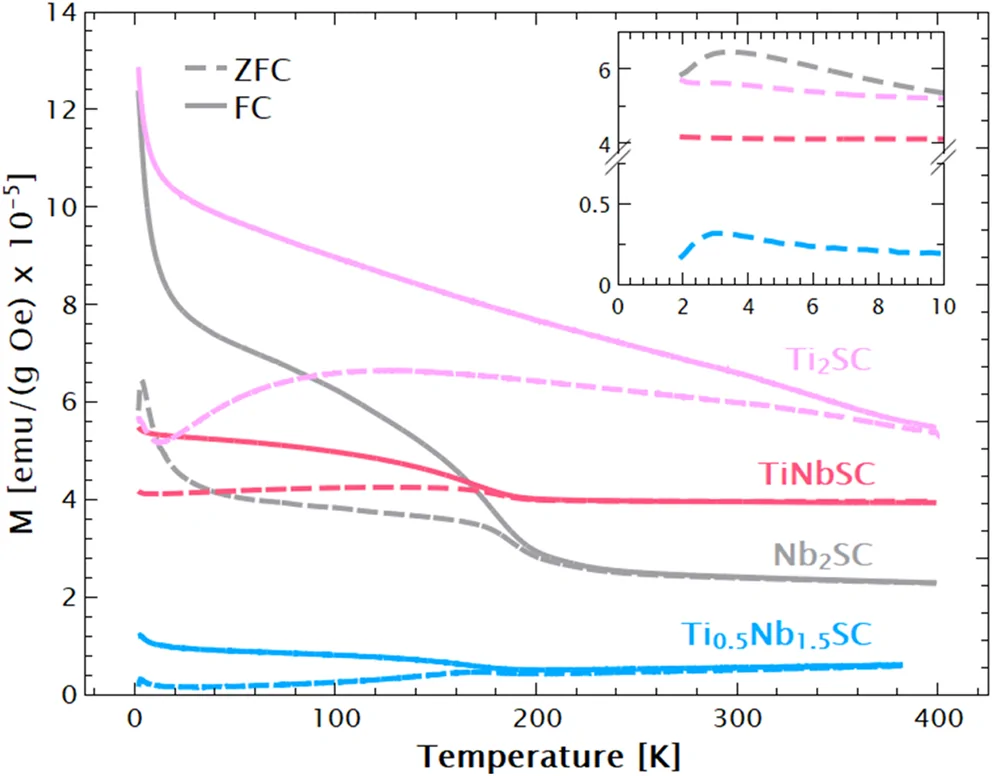
Temperature-dependent magnetization of Ti_*x*_Nb_2–x_SC. Nb_2_SC, Ti_0.5_Nb_1.5_SC, TiNbSC, and Ti_2_SC under field cooling and zero field cooling. The inset shows the ZFC curves in the temperature region 0–10 K.

From the totality of the bulk magnetic and room-temperature diffraction results, it appears that although the existence of iron was not observed in the EDS measurements, small amounts of ferromagnetic-containing impurities remain in all samples. This is further supported by the field-dependent magnetization measurements (c.f. Figure S3), where all samples at all temperatures show soft ferromagnetic behavior.^42^ Due to its ferromagnetic characteristics even at elevated temperatures (*T*_c_ = 1043 K), a minute amount can be responsible for a large magnetic signal. In this sense, the magnetization measurements serve as a very sensitive probe for the ability to wash off the iron used for the samples’ synthesis, as well as its byproducts.

To carefully study the low-temperature physical and crystallographic properties of Ti_*x*_Nb_2–*x*_SC, methods were needed to distinguish the main studied phases from the impurities. Neutron diffraction as a function of temperature is one such method. Neutron diffraction enables us to simultaneously study crystal symmetry, content, and dimension, and whether any of its constituents order magnetically.

The 10 K neutron diffraction measurements of samples with *x* = 0 and 0.5, taken on KANDI-II, show no additional reflections and no intensity increase of select reflections when compared to the 300 K data (Figure 2). In addition, Rietveld analysis agrees excellently with neither spontaneous magnetic ordering nor a crystallographic transition in these phases. The high-resolution [on Echidna (Figure S4)], and high-intensity [on Wombat (not shown)] temperature-dependent NPD measurements of Nb_2_SC show an indication of neither spontaneous magnetic ordering nor a crystallographic transition as well, supporting the above-mentioned result for this phase. Lattice parameters of the base temperatures (10 K for KANDI-II, 3 K for Echidna, and 4 K for Wombat) determined by Rietveld refinement analysis are summarized in Table 1 and Figure S2.

Lattice parameters of Ti_*x*_Nb_2–*x*_SC (*x* = 0, 1, 2) MAX phase behavior as a function of temperature, determined by NPD measurements on Wombat, show no special anomaly, in agreement with the observation that there is neither magnetic nor crystallographic transition in these phases between 2 and 370 K (Figure 5). Data were analyzed using a model that combines the electronic and lattice contributions to the thermal expansion [eq 1],^43,44^

1 $$L\left(T\right)=L_{0}+A_{el}T^{2}+3NRA_{lat}\left[{3\mathrm{Tw}}_{D}{\left(\frac{T}{\theta_{D}}\right)}^{3}\int_{0}^{\theta_{D}/T}\frac{x^{3}}{e^{x}-1}\mathrm{dx}+\left(1-w_{D}\right)\frac{\theta_{E}}{e^{\theta_{E}/T}-1}\right]$$

**Figure 5:**
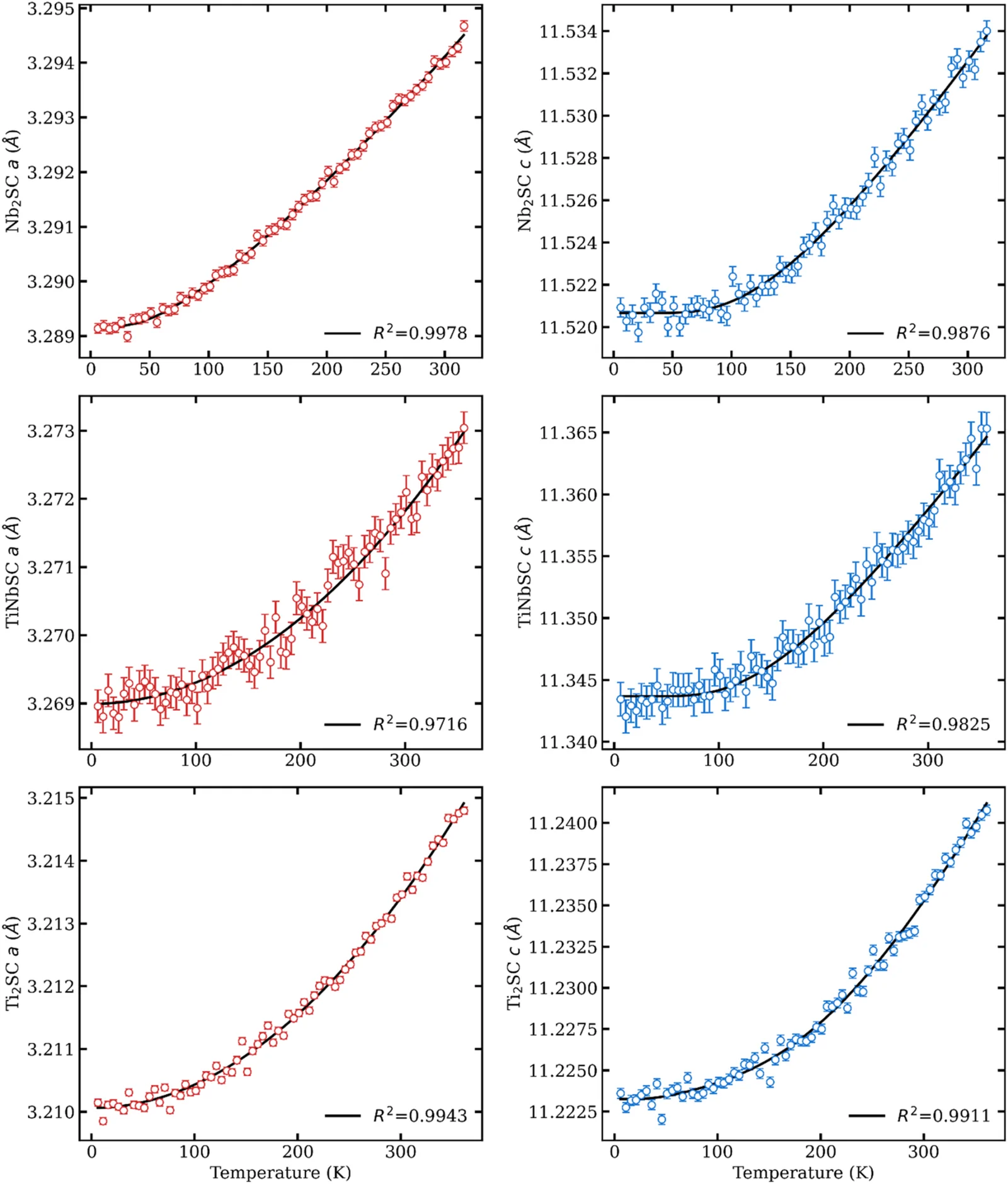
Thermal expansion of Nb_2_SC (top row), TiNbSC (middle row), and Ti_2_SC (bottom row). Lattice parameters *a* (left column) and *c* (right column) are determined using Rietveld analysis of NPD measurements taken on Wombat (open circles). “Constrained” thermal expansion model, based on eq 1, is fitted to the data (solid line).

In this model, *L*(*T*) is the temperature-dependent lattice parameter being analyzed (*a*, *c*, or *V* ^1/3^); *L*_0_ is its value at zero temperature; *A*_el_ is the electronic expansion coefficient that represents the contribution of the free electron gas to the expansion; *A*_lat_ is the lattice expansion coefficient that represents the changes in the lattice vibrational energy; w_D_ is the Debye model contribution to the thermal expansion contributed by the lattice vibrations, while (1 – *w*_D_) represents the respective Einstein model contribution; *N* = 4 is the number of atoms per formula unit; and *R* = 8.3145 J/(mol K). Fitting this model to the experimental results enables the estimation of Debye, θ_D_, and Einstein, θ_E_, temperatures, as well as the specific character of the expansion along the different axes directions.

When allowing all parameters of eq 1 to be refined (an unconstrained model), the process resulted in extremely large uncertainties in select fitted parameters, often eclipsing the refined values. Therefore, additional reasoning was used in the fitting process. A closer look at the *a*-axis thermal expansion of the three samples shows that they have a near-pure quadratic form, indicating a significant electronic term. In contrast, it is obvious that the *c*-axis thermal expansion exhibits, especially for Nb_2_SC and TiNbSC, a significantly large low-temperature range where almost no thermal expansion is observed (Figure 5), indicating a negligible electronic term compared to other terms. Sulfur is a highly electronegative chalcogen. It localizes electrons into strong covalent/ionic M–S bonds, lacking the free electron sea required for metallic transport. This chemical distinction restricts the metallic charge carriers almost exclusively to the M–X basal planes, yielding strongly anisotropic electrical conductivity and a quasi-2D Fermi surface.^45,46^ Therefore, the *a*-axis expansion is postulated to be driven by a combination of 2D electronic pressure (*A*_el_ > 0) and basal plane phonons. Conversely, the *c* axis spans the nonmetallic M–S gaps, and its expansion is assumed to be driven almost entirely by lattice anharmonicity (*A*_el_ ≈ 0) and a different mixture of acoustic and optical phonons. Therefore, for each direction and sample, 4 models were fitted to the data: 1: unconstrained; 2: electronic and Debye terms (*w*_D_ = 1); 3: electronic and Einstein term (*w*_D_ = 0); and 4: “constrained”. The “constrained” model was chosen in each case according to the above-mentioned physical reasoning, the stability of the fit of the first three models, the values’ uncertainties, and their goodness of fit. This is the model presented here (Table 2 and Figure 5). Results of all models are presented in Table S2. It is worth noting that the unconstrained model for all samples results in very high and nonphysical uncertainties for several fitted parameters, indicating overfitting to the data using this model.

**Table 2:** Fit Parameters of a “Constrained” Physical Thermal Expansion Model to the Lattice Parameters of Ti_*x*_Nb_2–*x*_SC with *x* = 0, 1, 2 as a Function of Temperature Measured Using Neutron Diffraction on Wombat [eq 1]­^a^

compound		*L*_0_ (Å)	*A*_el_	*A*_lat_	θ_D_ (K)	θ_E_ (K)	*w*_D_
Nb_2_SC	*a*	3.28916(3)	13(5)	0.17(3)	228(31)	[0]	[1]
	*c*	11.5207(1)	[0]	0.87(4)	[0]	422(22)	[0]
TiNbSC	*a*	3.26899(4)	31.4(7)	[0]			
	*c*	11.3437(2)	[0]	1.3(1)	[0]	483(43)	[0]
Ti_2_SC	*a*	3.21006(1)	37.3(2)	[0]			
	*c*	11.2233(1)	[0]	1.5(1)	227(10)	860(104)	0.17(5)

a *L*_0_ is the zero-temperature lattice parameter, *A*_el_ is the electronic expansion coefficient, *A*_lat_ is the lattice expansion coefficient, θ_D_, and θ_E_ are the Debye and Einstein temperatures, respectively, and *w*_D_ is the Debye model contribution. Parentheses represent fit uncertainty of the last significant digit. Square parentheses represent fixed values in the model.

The thermal expansion results support the strong anisotropy of the Ti_*x*_Nb_2–*x*_SC system. As mentioned above, the expansion in the basal plane is governed by the electronic pressure of the metallic layer. In contrast, the thermal expansion across the layers through the nonmetallic Ti–S bonds can be excellently explained with no electronic term at all. Moreover, these results can be explained mostly by Einstein’s optical modes. The nature of the lattice vibrations along the nonmetallic gap varies dramatically with mass. In pure Nb_2_SC, the extreme mass contrast between the heavy Nb_2_C block and the light S atoms creates a large acoustic impedance mismatch. Long-range acoustic waves cannot efficiently couple across this gap, forcing the S atoms to vibrate independently in localized optical (Einstein’s) modes.^43^ In pure Ti_2_SC, substituting the lighter Ti atoms reduces this acoustic impedance mismatch, allowing a small fraction of low-frequency acoustic phonons to couple across the gap [yielding *w*_D_ = 0.17(5); Table 2]. However, in the TiNbSC solid solution, the mass-variance and static strain fields within the alloyed M-layer reintroduce intense point-defect scattering of phonons.^47^ This scattering destroys the acoustic wave translation seen in Ti_2_SC, causing the phonons to localize into flat-band vibrations.^48^ As a result, the TiNbSC *c*-axis expansion collapses back entirely to localized rattling modes, and can be modeled using *w*_*D*_ = 0.

The physical behavior of thermal expansion can help understand the anisotropic relative thermal strain observed in the studied samples (Figure 6). Cooling from room temperature (296 K) down to 2 K, opposite trends are observed when comparing the three phases. Nb_2_SC contracts ∼50% more than Ti_2_SC and TiNbSC along the hexagonal plane, while out of the hexagonal plane it is the equiatomic solid-solution (*x* = 1) that contracts ∼35% more than both end compounds (*x* = 0, 2). These opposite trends resulted in a total volume contraction of ∼30% more for Nb_2_SC compared to Ti_2_SC and TiNbSC. Interestingly, and probably counterintuitive, TiNbSC’s volumetric contraction as a function of temperature is the smallest of the three that were studied by temperature-dependent NPD. These macroscopic observations could be explained by the microscopic thermodynamic decoupling modeled in this work. First, the bulk thermodynamic dilation of an anisotropic crystal is governed by the trace of its thermal expansion tensor.^49^ For a hexagonal lattice, this geometric trace yields a total volumetric expansion that is mathematically dominated by the basal plane, since β­(*T*)** = 2α_a_(*T*) + α_c_(*T*), where β­(*T*) is the volumetric thermal expansion and α_a_ (α_c_) is the *a* (*c*) axis thermal expansion. Therefore, the *a*-axis behavior primarily dictates the bulk volume. Second, it is hypothesized that the ∼50% *a*-axis overcontraction in Nb_2_SC occurs because the thermal expansion/contraction in this direction is driven by both electronic and vibrational inflation/deflation (Figure 5 and Table 2). In contrast, the TiNbSC basal plane is governed by positive chemical pressure, which elastically hardens the M_2_C block. Combined with the complete structural scattering of acoustic phonons, discussed in the previous paragraph, TiNbSC shrinks solely via the loss of 2D electronic pressure. Because the mathematically dominant *a*-axis is relatively anchored in TiNbSC, its total volumetric contraction is heavily restricted. Conversely, the anomalous ∼35% overcontraction of the TiNbSC *c*-axis highlights severe solid-solution frustration. As mentioned above, expansion across this nonmetallic gap is driven entirely by S-layer rattling. The mismatched Ti and Nb atomic radii probably create large static strain fields, forcing the compliant S atoms into highly asymmetric, anharmonic regions of their potential wells.^50^ High anharmonicity directly yields a massive localized Grüneisen parameter.^51^ When this highly anharmonic system is cooled to 2 K, the thermal rattling pressure vanishes rapidly, causing the nonmetallic gap to collapse drastically compared to the symmetric pure phases. However, since this *c*-axis collapse contributes to the volume linearly, it is mathematically overpowered by the rigid *a*-axis, with good agreement with TiNbSC being the least contractive material overall.

**Figure 6:**
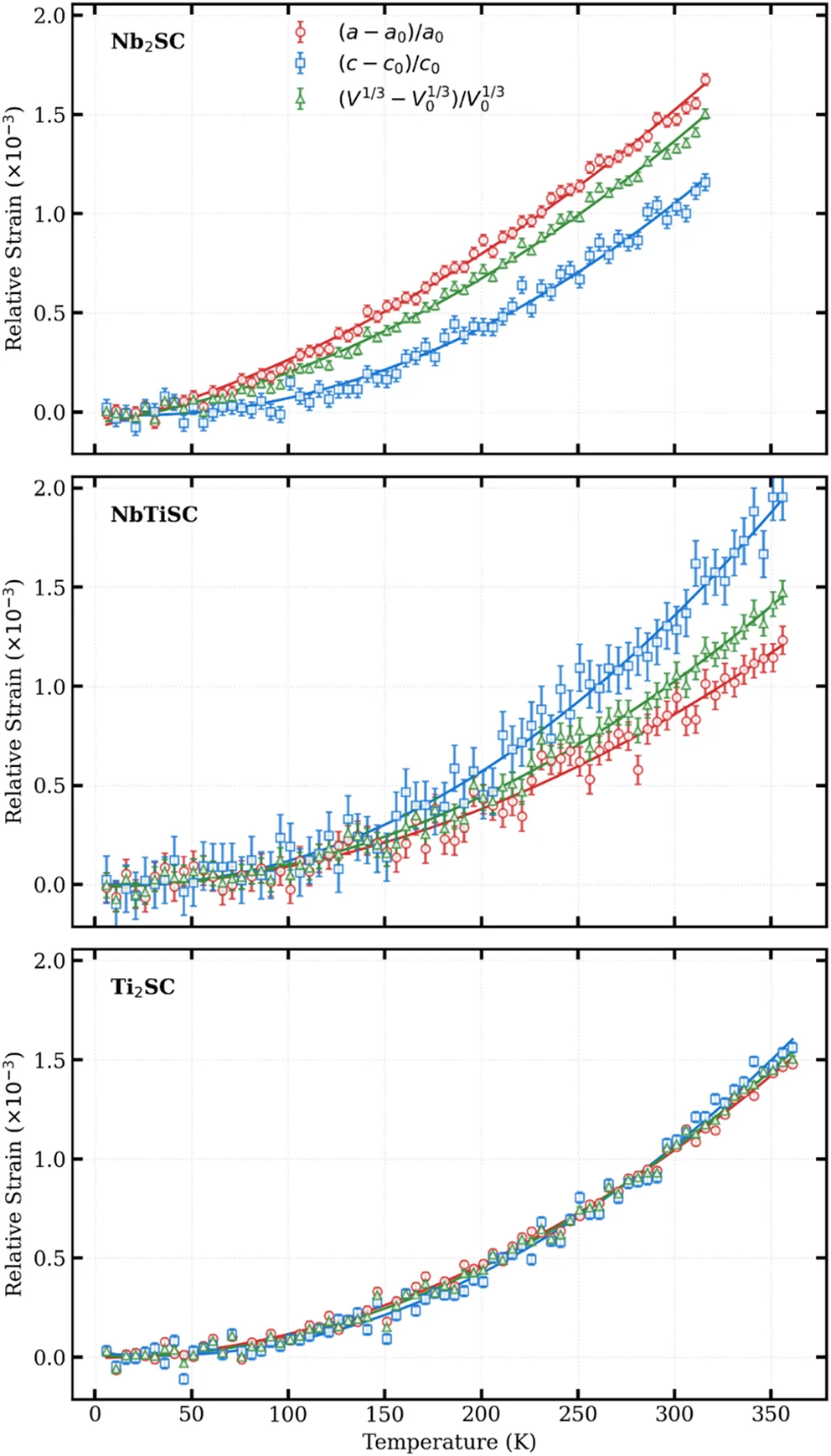
Temperature dependency of the anisotropic relative thermal strain of Nb_2_SC, TiNbSC, and Ti_2_SC. The relative thermal strain was calculated with $\frac{L-L_{0}}{L_{0}}$ using the refined lattice parameters with *L* equal to *a*, *c*, or *V* ^1/3^ (cube root of cell volume) of Nb_2_SC, TiNbSC, and Ti_2_SC, determined using Rietveld analysis of NPD measurements taken on Wombat. *L*_0_ represents the lattice parameter at 0 K extracted from fitting eq 1 to the thermal expansion data. The trend lines are a guide to the eye.

The constant-pressure heat capacity profiles as a function of temperature of Ti_*x*_Nb_2–*x*_SC (*x* = 0, 0.5, 1, 1.5, 2) samples, *c*_p_(*T*), show no signature of phase transition in the measured temperature ranges (Figure 7). The solid solutions’ specific heat curves appear between the two end compounds, as expected. All measured curves approach an asymptotic behavior limited by ∼100 J/(mol K), in agreement with the Dulong-Petit law, in which at *T* → ∞, the constant-volume specific heat, *c*_v_ = 3*NR* ≅ 100 J/(mol K).^52^ *c*_v_(*T*) is expressed in terms of the Debye–Einstein model [eq 2] ^52,53^ summed with an electronic contribution proportional to the Sommerfeld coefficient, γ. Fitting [eq 2] to the experimental profiles yields θ_*D*_ and θ_*E*_ temperatures, as well as γ for each sample (Table 3). All fits considered the experimental uncertainties and were constrained by the Dulong–Petit law.

2 $$c_{V}=\gamma T+9\mathrm{NR}w_{D}{\left(\frac{T}{\theta_{D}}\right)}^{3}\int_{0}^{\theta_{D}/T}\frac{x^{4}e^{x}}{{\left(e^{x}-1\right)}^{2}}\mathrm{dx}+3\mathrm{NR}\left({1-w}_{D}\right)\frac{{\left(\frac{\theta_{E}}{T}\right)}^{2}e^{\theta_{E}/T}}{{\left(e^{\theta_{E}/T}-1\right)}^{2}}$$

**Figure 7:**
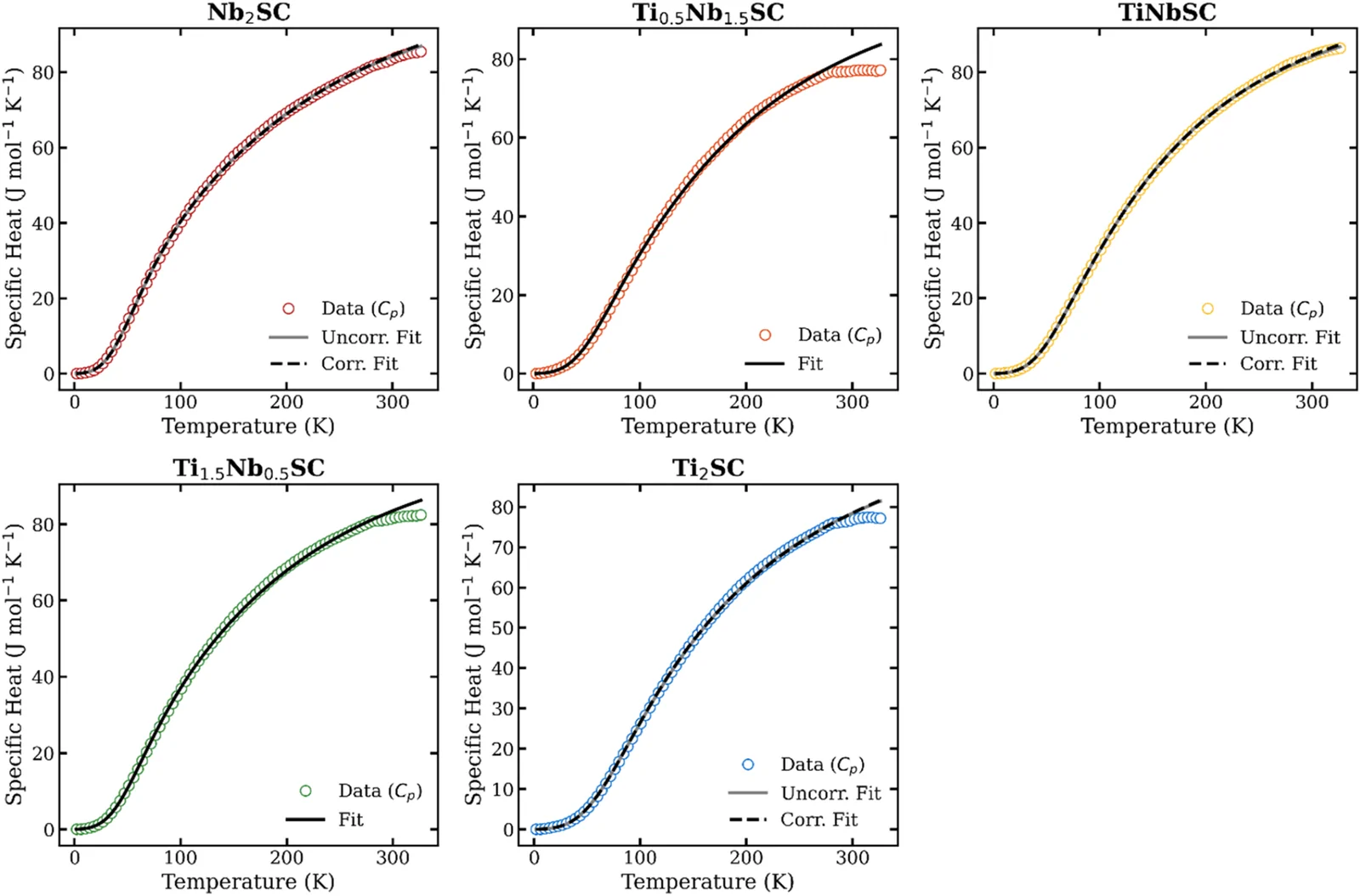
Heat capacity of Ti_*x*_Nb_2–*x*_SC measured (circles) temperature scans of the constant-pressure heat capacity of samples with *x* = 0, 0.5, 1, 1.5, and 2. A Debye–Einstein model [eq 2] was fitted to the measured results (solid lines). A model, corrected for dilation, is fitted to results of samples with *x* = 0, 1, 2 (dashed lines; see text).

**Table 3:** Fitted Debye (θ_D_) and Einstein (θ_E_) Temperatures Using the Debye-Einstein Model [eq 2]­^a^

sample	θ_D_ (K)	θ_E_ (K)	γ [mJ/(mol K^2^)]	*w*_D_
Nb_2_SC	341.2(4)	807(3)	12.56(3)	0.646(1)
Ti_0.5_Nb_1.5_SC	445.0(1)	825(3)	10.00(4)	0.656(2)
TiNbSC	436.0(1)	718(3)	9.42(4)	0.676(3)
Ti_1.5_Nb_0.5_SC	386.2(4)	826(2)	12.00(1)	0.675(1)
Ti_2_SC	532.0(1)	958(6)	6.38(4)	0.773(2)

a Sommerfeld coefficient, γ, and the Debye part relative contribution, *w*_D_, are given too. Numbers in parentheses represent one standard deviation of the values determined from the fitting process, and refer to the last significant digits. For all fits, the goodness of fit *R* ^2^ > 0.995.

Note that the Debye–Einstein model holds for constant-volume specific heat, *c*_v_, while usually the constant-pressure specific heat, *c*_p_, is measured. However, for solids the volumetric thermal expansion is relatively very small, especially at low temperatures (e.g. Figure 6). Therefore, *c*_p_ ≈ *c*_*v*_ is a very good approximation. Having said that, *c*_p_ can be modeled by adding the thermodynamic dilation correction (*c*_dil_) to *c*_v_,^49^ where

3 $$c_{dil}=V_{m}TB_{T}\beta {\left(T\right)}^{2}$$

with *V*_m_ being the molar volume, and *B*_T_ the isothermal bulk modulus. *c*_dil_ can be calculated for Ti_*x*_Nb_2–*x*_SC, with *x* = 0, 1, 2, using the thermal expansion fits done in this work. For TiNbSC, we used an averaged bulk modulus, 194 GPa, calculated from the measured bulk modulus of Ti_2_SC [191(3) GPa][Bibr ref15] and the bulk modulus of Nb_2_SC (∼197 GPa)^55^ estimated from calculations.

As expected, the corrected fits show almost no change compared with the uncorrected ones (Figure 7). Consequently, the refined parameters show only minor deviations. Since no thermal expansion measurements were done for the *x* = 0.5 and 1.5 samples, the corrected model cannot be used for all studied samples. Therefore, refined parameters from the specific heat fits presented here are those for the uncorrected model [eq 2 and Table 3].

The thermodynamic properties of Nb_2_SC and Ti_2_SC were previously studied by theory and experiment, respectively.^16,55,56^ The Ti_2_SC specific heat measured here is in agreement with the experimental results published previously.^16,56^ However, the Debye temperature refined here, using eq 2, is significantly smaller than the previously reported.^16,56^ Note that the models used in refs 16 and 56 apply for low temperatures, in the region of *T* < θ_D_/50 (i.e., *T* < 15 K for the result reported there).^52^ In particular, equation 11 in ref 16 is determined from the Debye model at very low temperatures,^52^ while the Debye temperature determined in this work is a result of a fit up to ∼300 K. Moreover, the fit in the present work is constrained to the Dulong-Petit limit, which prevents artificial inflation of the Debye temperature.

To date, the specific heat of Nb_2_SC has not been measured. *Ab initio* calculations of the Nb_2_SC specific heat resulted in an estimation of θ_D_ ∼ 540 K at ambient pressure,^55^ larger than what is reported here (341 K). However, the model used there did not consider an Einstein’s term, which is quite common in the analysis of low-temperature specific heat data. In addition, the calculated result was made assuming a 0 K quasi-harmonic model^55^ that fails to capture the anharmonicity introduced by the S-layers rattling at finite temperatures.

The refined parameters (Table 3) demonstrate significant deviation from the Kopp-Neumann rule. This rule predicts monotonous evolution of θ_D_ for the solid solutions between the two end compounds, following the inverse square root of the average mass. We propose that this deviation is driven by a synergistic mechanism involving both localized static strain and alterations to the valence electron concentration (VEC). Ti (Group IV) and Nb (Group V) differ significantly in both atomic radius and valence electron count. In the M-X rocksalt blocks of MAX phases, altering the transition metal VEC fundamentally modifies the macroscopic elasticity. It is established that increasing the VEC in transition metal carbides populates the d-t_2g_ orbitals, which overlap upon shear and degrade the shear modulus (C_44_), leading to macroscopic lattice softening.^57^

On the Nb-rich side, in Ti_0.5_Nb_1.5_SC, the substitution of smaller Ti atoms into the larger Nb matrix induces positive internal chemical pressure. Concurrently, the substitution of a Group 4 element into a Group 5 matrix decreases the overall VEC, thereby depleting the destabilizing d-t_2g_ orbitals and enhancing the shear resistance of the M–C bonds. The combination of local steric contraction and electronic bond stiffening steepens the interatomic potential well, substantially increasing the effective macroscopic spring constant (*k*). Coupled with the reduction in average atomic mass, these synergistic structural and electronic effects provide a physical interpretation for the pronounced elevation of θ_D_ to 445 K.

Conversely, on the Ti-rich side, in Ti_1.5_Nb_0.5_SC, the substitution of larger Nb atoms into the tightly packed Ti matrix induces significant localized static expansion. Simultaneously, the introduction of the Group 5 element increases the overall VEC, populating the d-t_2g_ orbitals and electronically degrading the shear modulus. In the framework of anharmonic lattice dynamics, the localized static expansion forces the interatomic bonds outward into the shallower regions of their asymmetric potential wells. This static displacement degrades the effective macroscopic spring constant.^50^ When it operates in tandem with the electronic softening induced by the altered d-band filling, this suggested mechanism accounts for the pronounced, anomalous softening of the lattice (dropping θ_D_ to 386 K), completely overriding the Kopp-Neumann mass-averaging rule.

When comparing the Debye and Einstein temperatures extracted from the bulk specific heat to those derived from the directional thermal expansion (Tables 2 and 3), it cannot be assumed that the *c*_p_ results are a simple spatial average of the directional fits. This discrepancy arises from the fundamentally different ways these two macroscopic properties sample the microscopic phonon density of states across the Brillouin zone. Specific heat is a purely scalar thermodynamic quantity. It integrates the internal energy of all vibrational modes purely based on their frequency distribution, regardless of their structural anharmonicity. In contrast, directional thermal expansion acts as a highly selective physical filter.^51^ It isolates only the subset of phonon modes that possess a nonzero, direction-specific mode-Gruneisen parameter. For example, an in-plane vibrational mode may possess high energy (significantly contributing to the bulk *c*_p_ and raising the bulk θ_D_) but possesses a near-zero mode-Gruneisen parameter (contributing nothing to the *a*-axis thermal expansion, as seen above for TiNbSC). Consequently, the directional thermal expansion fits map structurally active anharmonicity, while the *c*_p_ fits map the total harmonic energy of the lattice, ruling out a simple 2:1 mathematical averaging between the axes.

Since specific heat is an extensive thermodynamic property, the measured *c*_p_(*T*) profiles represent a mass-weighted sum of the main phase and the secondary impurity phases. Notably, Nb_2_SC contains the largest impurity fraction among the studied samples (∼10 wt % combined impurities, primarily Fe_2_Nb and NbC). Consequently, it was analyzed as a limiting case to estimate the maximum deviation in specific heat induced by secondary phases. The extracted parameters for the Nb_2_SC composite [θ_D_ = 341.2(4) K and γ = 12.56(3) mJ/(mol K^2^)] represent effective macroscopic values. To establish the thermodynamic bounds of the pristine phase, the specific heat parameters of the primary impurities were utilized. The literature values for NbC_0.93_ are θ_D_ = 496(10) K and γ = 3.2 mJ/(mol K^2^).^58^ Furthermore, recent thermodynamic modeling of the C14 Laves phase Fe_2_Nb establishes its parameters as θ_D_ = 532 K and γ = 19.8 mJ/(mol K^2^).^59^ Applying a mass-weighted Kopp-Neumann correction to the low-temperature regime demonstrates that subtracting these structurally stiffer impurity contributions further lowers the calculated intrinsic Debye temperature of the pristine Nb_2_SC phase to ∼333 K, while the Sommerfeld coefficient is marginally adjusted to ∼12.7 mJ/(mol K^2^). Therefore, the presence of impurities does not artificially induce the observed lattice softening. Rather, the low θ_D_ driven by the anharmonicity of the nonmetallic S-layers is robust against impurity-induced inflation. Nevertheless, further thermodynamic investigations on higher-purity samples are required.

## 4 Conclusion

Nb_2_SC, Ti_2_SC, and their solid solutions Ti_*x*_Nb_2–*x*_SC (*x* = 0.5, 1, 1.5) were successfully synthesized by a one-step reaction under Ar using FeS as an effective sulfur source, enabling the rapid and scalable synthesis of S-based MAX phases. Although most of the Fe was removed through HCl treatment, magnetization measurements, supported by X-ray and neutron diffraction, revealed trace Fe-based impurities that remain detectable.

TGA showed that Nb-rich phases, i.e., Nb_2_SC and Ti_0.5_Nb_1.5_SC, oxidize near ∼480 °C, whereas Ti-rich phases, i.e., TiNbSC, Ti_1.5_Nb_0.5_SC, and Ti_2_SC, exhibit substantially higher stability. Oxidation of Nb_2_SC at 750 °C in air produced a sulfur-bearing niobium oxide with a layered morphology, suggesting a simple pathway to sulfur-modified layered mixed oxides directly from carbosulfide MAX precursors.

Low-temperature magnetic measurements of Nb_2_SC and Ti_0.5_Nb_1.5_SC revealed a sharp ZFC downturn, which hints at superconductivity. However, an absolute negative magnetic response was never observed down to 2 K, and the lack of a corresponding λ anomaly in the specific heat data confirms the absence of bulk superconductivity. This partial diamagnetic shielding is attributed to trace, interconnected superconducting impurities (such as Nb_2_S_2_C) whose bulk thermodynamic signal is negligible, together with the masking effects of the highly magnetic Fe-based impurities. It is important to keep in mind that using FeS as an agent for rapid, large-scale S-based MAX phase manufacturing may affect properties sensitive to ferromagnetic impurities.

Substituting Nb atoms with Ti led to a linear decrease in both lattice parameters, with slightly stronger contraction along the *c* axis (∼3.1%) than the *a* axis (∼2.8%). This anisotropy in lattice dimensions decreases between Nb_2_SC and Ti_2_SC corroborating the strong anisotropy observed in the thermal expansion. The latter is explained in the framework of the difference between the primarily metallic in-plane bonds and nonmetallic out-of-plane ones. In the solid solutions, when mixing effects are involved as well, a much more complex behavior is observed, involving significant phonon scattering and high anharmonicity that act as competing mechanisms in the directional thermal expansion, resulting in TiNbSC having smaller volumetric thermal expansion compared to the end members.

Specific heat measurements as a function of temperature indicate that bulk Ti_2_SC is stiffer than bulk Nb_2_SC. The specific heat curves of the solid solutions lie between those of the end members but do not follow the Kopp-Neumann rule owing to substantial localized strain fields introduced by Ti/Nb mixing. For the Ti-poor solid solutions, this effect further adds to the stiffness associated with the reduced average atomic mass upon substitution of Nb with Ti. In contrast, for the Ti-rich solid solutions, it substantially suppresses the mass-driven increase in the stiffness.

Furthermore, the ability to tune the valence electron concentration in the Ti_*x*_Nb_2–*x*_SC system holds significant promise for their 2D derivatives. When MAX phases are exfoliated into 2D MXenes, the ’A’ element is etched away, leaving transition metal carbide layers with highly electronegative surface terminations. As highlighted by previous studies on 2D transition metal compounds with strongly electronegative surfaces,^60^ such architectures possess diverse charge-donating and accepting abilities that are highly advantageous for charge transfer, adsorption, catalysis, and energy storage applications. The capacity to fine-tune the Ti/Nb ratio, and consequently the d-band filling, provides a fundamental lever to optimize the core electronic properties of these future 2D MXenes for targeted nanoscale applications.

Overall, layered carbosulfide MAX phases exhibit pronounced directional anisotropy arising from distinct and sometimes competing physical mechanisms. Understanding these mechanisms is the key to fine-tuning macroscopic properties of interest in solid solutions of this family of compounds.

## Supplementary Material



## Notes

Molar ratios used to synthesize Ti_*x*_Nb_2–*x*_SC solid solutions; fit parameters of the physical thermal expansion model to the lattice parameters of Ti_*x*_Nb_2–*x*_SC, as a function of temperature measured using neutron diffraction on Wombat; energy-dispersive X-ray spectroscopy of Nb_2_SC treated at different etching parameters to remove Fe impurities; lattice parameters of Ti_*x*_Nb_2–*x*_SC determined from Rietveld analysis of XRD, and NPD data; field-dependent magnetization of Nb_2_SC; and high-resolution NPD profile of Nb_2_SC at 3, 6, 11, 50, and 100 K. (PDF)
