# Supplementary material for: Rapid and Scalable Synthesis, Structure, and Physical Properties of Solid-Solution Layered Titanium Niobium Carbosulfide “Ti x Nb2–x SC”
Source: ACS Omega. 2026 Sep 23;11(39):60388–400. doi: 10.1021/acsomega.6c08457 (PMC13647327; doi:10.1021/acsomega.6c08457)
Supplement: Supplementary file 1 [file ao6c08457_si_001.pdf]

Supporting Information

Rapid and Scalable Synthesis, Structure, and Physical Properties of Solid-Solution Layered Titanium Niobium Carbosulfide "$Ti_xNb_{2-x}SC$"

Ahmad Majed,[a] *Asaf Pesach,*[b] *Anna Eyal,*[c] *Amit Keren,*[c] *Mina Saleh,*[a] *Kaitlyn Prenger,*[a] *El'ad N. Caspi,*[b,*] *Maxim Avdeev,*[d] *Michael Naguib*[a,*]

[a] Department of Physics and Engineering Physics, Tulane University, New Orleans, LA 70118, USA.

[b] Physics Department, Nuclear Research Centre – Negev, POBox 9001, 84190, Beer-Sheva, Israel

[c] Technion

[d] Australian Centre for Neutron Scattering, Australian Nuclear Science and Technology Organisation, Locked Bag 2001, Kirrawee DC, NSW 2232, Australia

[*]Corresponding Author E-mail: eladc@iaec.gov.il, naguib@tulane.edu

Table S1 Molar ratios are used to synthesize $Ti_xNb_{2-x}SC$ solid solutions.

**Target structure**	***x***	**Nb**	**NbC**	**Ti**	**TiC**	**FeS**
**Nb_2SC**	0.0	1	1	0	0	1
**$Ti_{0.5}Nb_{1.5}SC$**	0.5	0.75	0.75	0.25	0.25	1
**TiNbSC**	1.0	0.5	0.5	0.5	0.5	1
**$Ti_{1.5}Nb_{0.5}SC$**	1.5	0.25	0.25	0.75	0.75	1
**Ti_2SC**	2.0	0	0	1	1	1

Table S2 Fit parameters of the physical thermal expansion model to the lattice parameters of $Ti_xNb_{2-x}SC$ with x = 0, 1, 2 as a function of temperature measured using neutron diffraction on Wombat [Eq. (1) in the text. 4 ways of refinement are compared for each sample in each crystallographic direction. Parentheses represent fit uncertainty of the last significant digit. Square parentheses represent fixed values in the model.

Nb_2SC | *a*-axis Models

Model	L_0 (Å)	A_{el} (1e-9)	A_{lat} (1e-6)	θ_D (K)	θ_E (K)	w_D	R^2
Unconstrained	3.28929(4)	0.3(443)	0.5(6)	307(37)	1462(1756)	0.4(6)	0.9961
Debye+Elec	3.28916(3)	13(5)	0.17(3)	228(31)	[0]	[1.00]	0.9978
Einstein+Elec	3.28939(2)	55(8)	0.0(3)	[0]	2000.0(1)	[0.00]	0.9845
Constrained	3.28916(3)	13(5)	0.17(3)	228(31)	[0]	[1.00]	0.9978

Nb_2SC | *c*-axis Models

Model	L_0 (Å)	A_{el} (1e-9)	A_{lat} (1e-6)	θ_D (K)	θ_E (K)	w_D	R^2
Unconstrained	11.5206(2)	0.2(2236)	0.9(19)	536(71672)	559(17166)	0.7(2583)	0.9875
Debye+Elec	11.5206(2)	0(441)	0.9(3)	610(41)	[0]	[1.00]	0.9875
Einstein+Elec	11.5207(2)	0(37)	0.9(2)	[0]	422(25)	[0.00]	0.9876
Constrained	11.5207(1)	[0.0]	0.87(4)	[0]	422(22)	[0.00]	0.9876

TiNbSC | *a*-axis Models

Model	L_0 (Å)	A_{el} (1e-9)	A_{lat} (1e-6)	θ_D (K)	θ_E (K)	w_D	R^2
Unconstrained	3.2690(1)	21(17)	0.1(1783)	556(201)	1646.5(1)	1(2622)	0.9719
Debye+Elec	3.2690(1)	21(17)	0.1(1)	556(201)	[0]	[1.00]	0.9719
Einstein+Elec	3.2690(1)	31(3)	0.00(6)	[0]	949.0(1)	[0.00]	0.9716
Constrained	3.26899(4)	31.4(7)	[0.00]	[0]	[0]	[0.00]	0.9716

TiNbSC | *c*-axis Models

Model	L_0 (Å)	A_{el} (1e-9)	A_{lat} (1e-6)	θ_D (K)	θ_E (K)	w_D	R^2
Unconstrained	11.3432(4)	127(67)	0.4(16676)	862(1)	606(284)	0(3844)	0.9842
Debye+Elec	11.3432(4)	116(83)	0.6(6)	853(402)	[0]	[1.00]	0.9841
Einstein+Elec	11.3432(4)	127(67)	0.4(4)	[0]	606(284)	[0.00]	0.9842
Constrained	11.3437(3)	[0.0]	1.3(1)	[0]	483(43)	[0.00]	0.9825

Ti_2SC | *a*-axis Models

Model	L_0 (Å)	A_{el} (1e-9)	A_{lat} (1e-6)	θ_D (K)	θ_E (K)	w_D	R^2
Unconstrained	3.21008(3)	23(17)	0.3(3)	430(149)	1650(1500)	0.3(4)	0.9944
Debye+Elec	3.21006(2)	37(2)	0.00(2)	881(1)	[0]	[1.00]	0.9943
Einstein+Elec	3.21006(1)	37.1(8)	0.00(1)	[0]	906(1)	[0.00]	0.9943
Constrained	3.21006(1)	37.3(2)	[0.00]	[0]	[0]	[0.00]	0.9943

Ti_2SC | *c*-axis Models

Model	L_0 (Å)	A_{el} (1e-9)	A_{lat} (1e-6)	θ_D (K)	θ_E (K)	w_D	R^2
Unconstrained	11.2232(1)	97(62)	0.6(8)	871(3)	807(687)	0.02(363)	0.9910
Debye+Elec	11.2232(1)	85(14)	0.71(9)	1130(167)	[0]	[1.00]	0.9910
Einstein+Elec	11.2232(1)	98(11)	0.55(7)	[0]	804(106)	[0.00]	0.9910
Constrained	11.2233(1)	[0.0]	1.5(1)	227(110)	860(104)	0.17(5)	0.9911

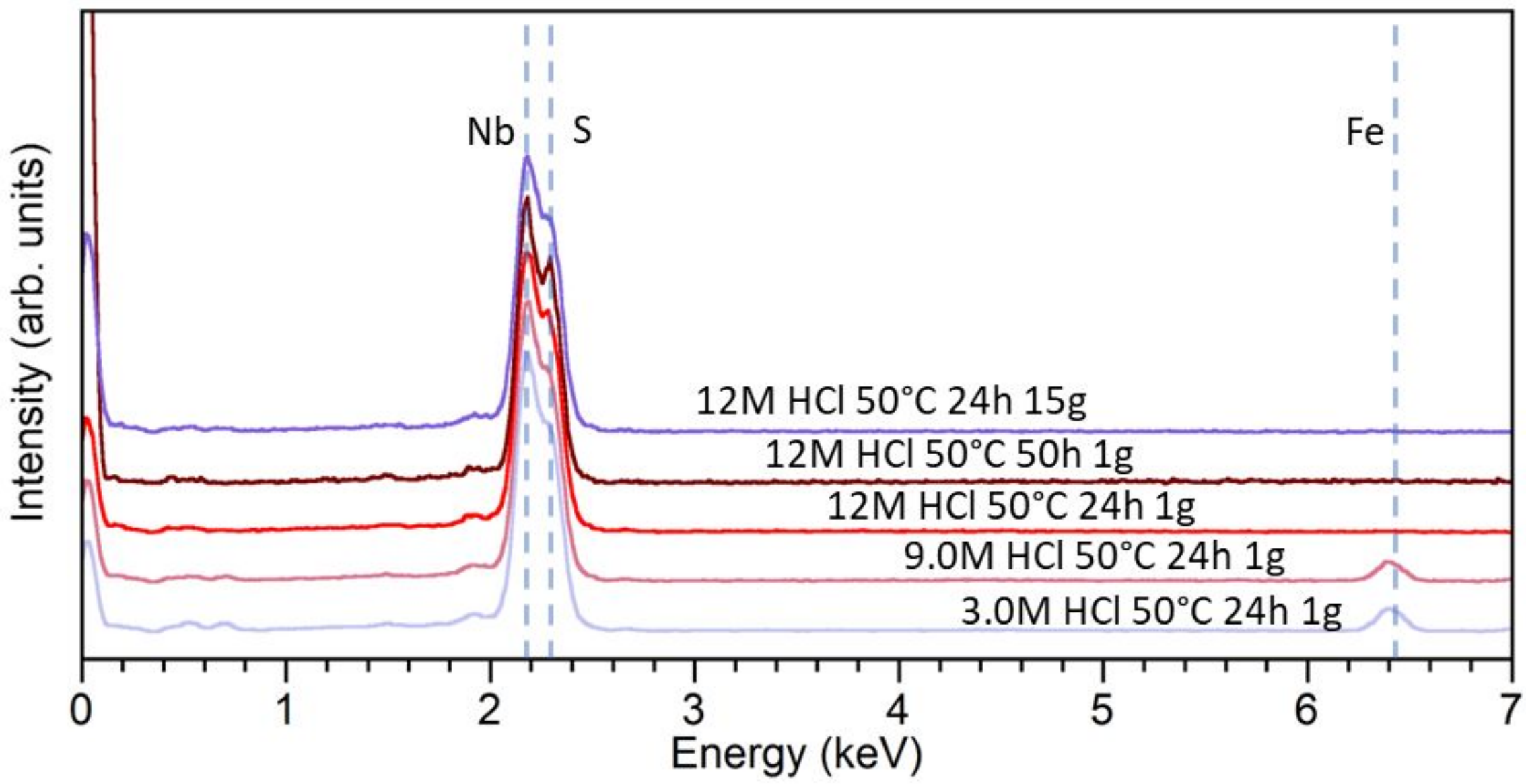

Figure S1 Energy dispersive X-ray spectroscopy of Nb_2SC treated at different HCl concentrations and durations at 50 °C.

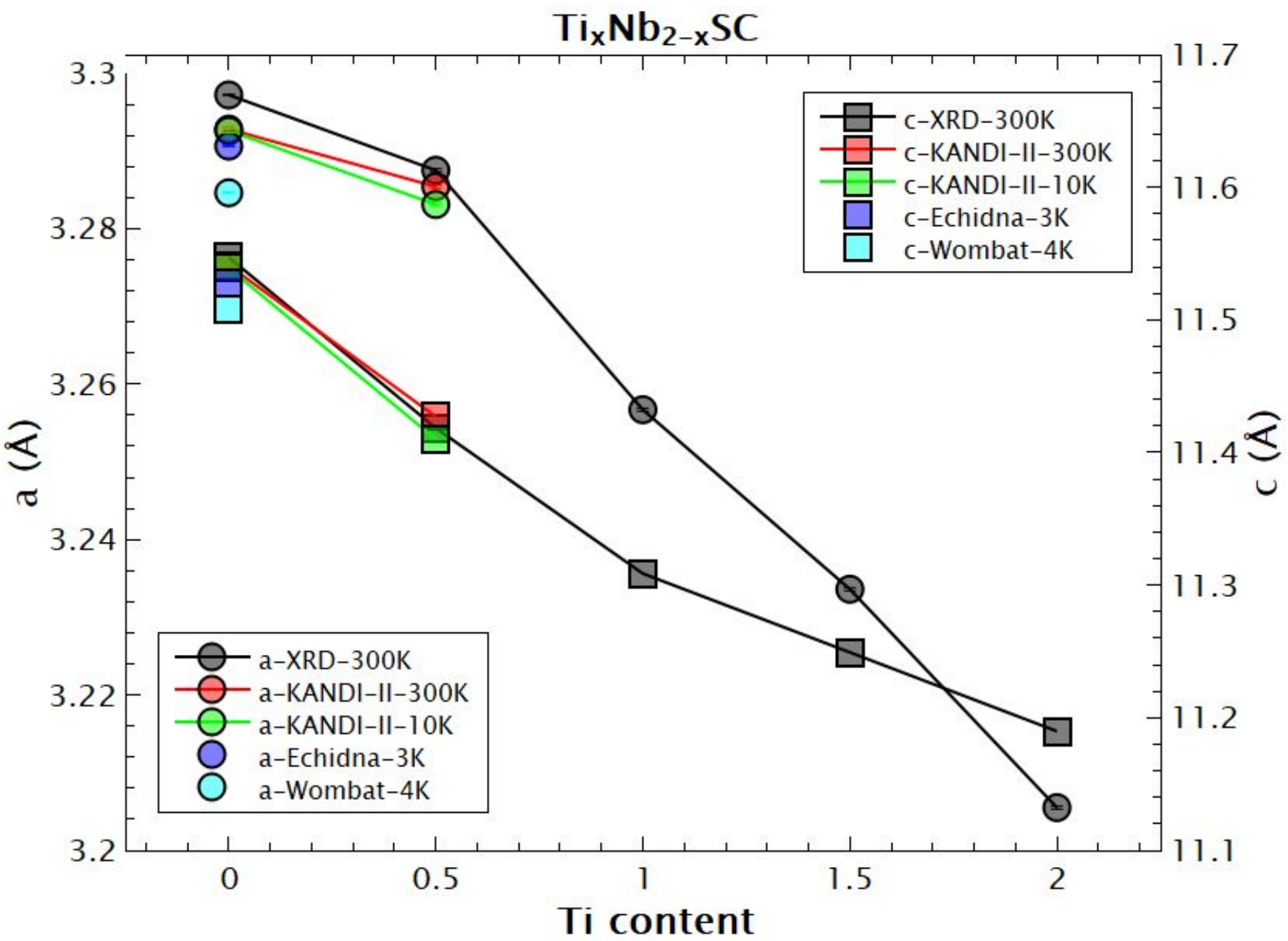

Figure S2 Lattice parameters of $Ti_xNb_{2-x}SC$ determined from Rietveld analysis of XRD, and NPD data. The NPD data is collected using the KANDI-II, Echidna, and Wombat diffractometers.

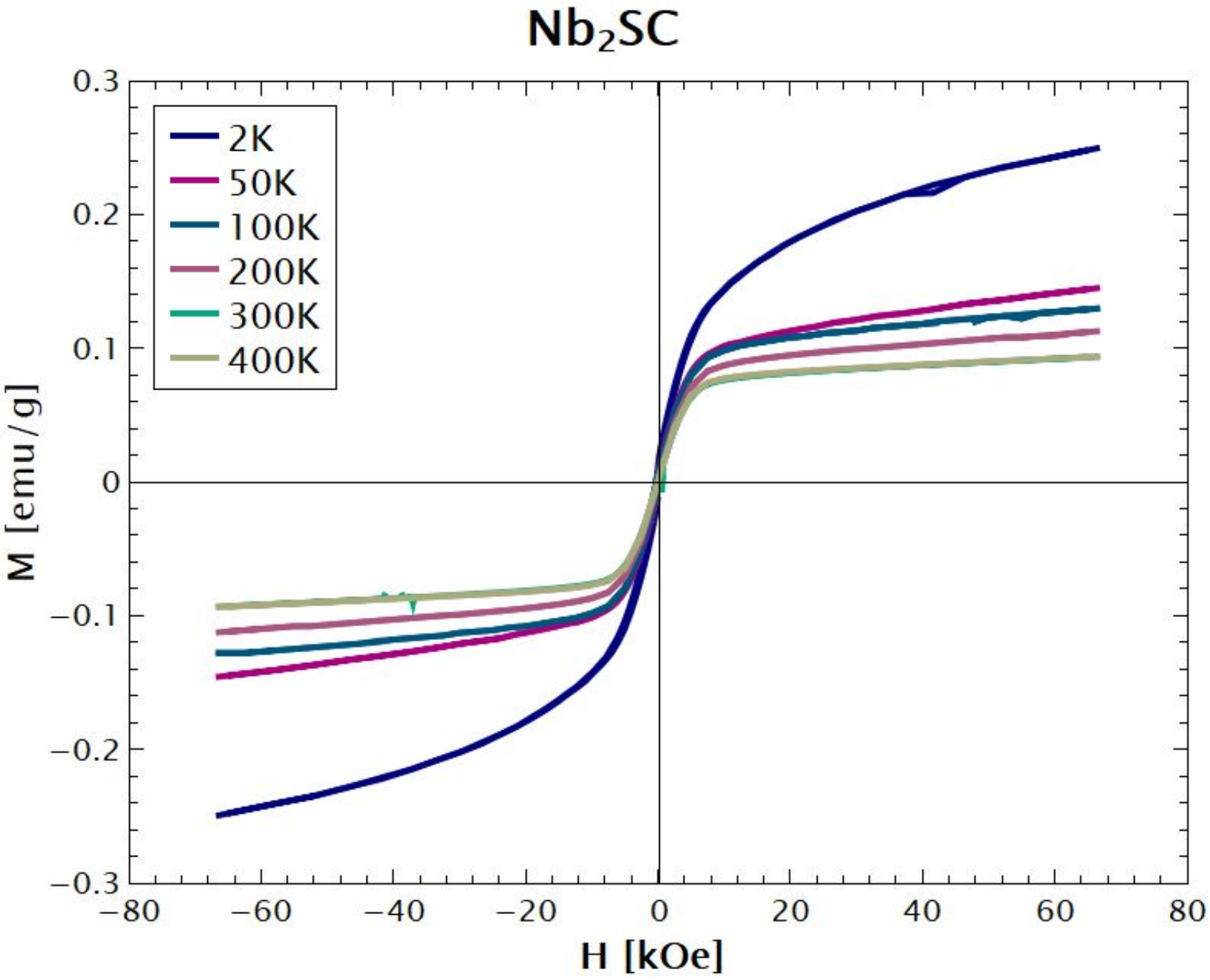

Figure S3 Field dependent magnetization of Nb_2SC: Measurements are taken in the 2 – 400 K temperature range. The 300, and 400 K curves are on top of each other.

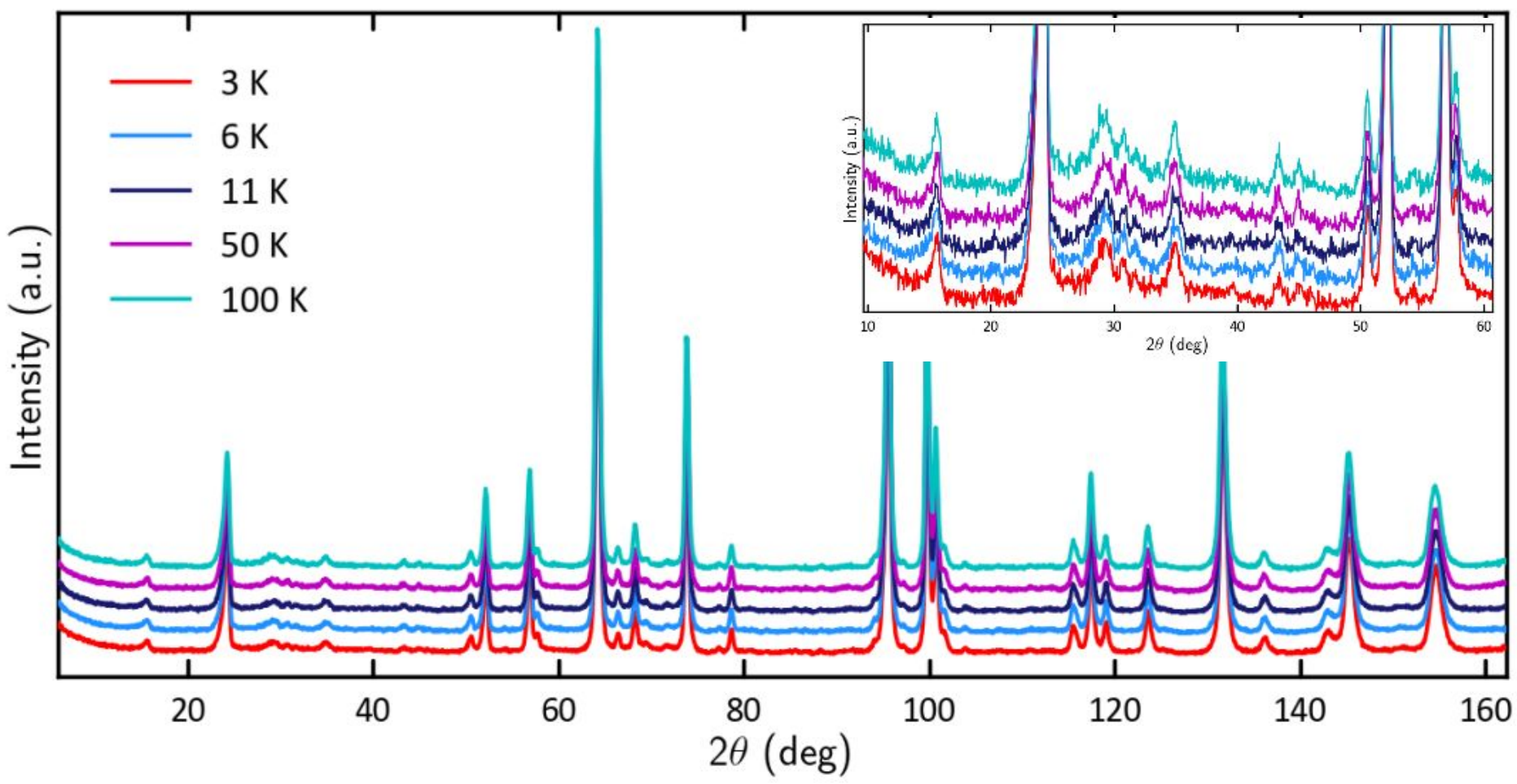

Figure S4 High-resolution NPD profile of Nb_2SC on Echidna: The measurements were recorded at five different temperatures: 3, 6, 11, 50, and 100 K. The observed NPD profiles are plotted (continuous lines) as a function of the scattering angle, 2θ. Inset: A zoom-in to the diffraction profile region between approximately 10° and 60°.
